# Ameliorative Effect of Sinapic Acid on Dextran Sodium Sulfate- (DSS-) Induced Ulcerative Colitis in Kunming (KM) Mice

**DOI:** 10.1155/2020/8393504

**Published:** 2020-11-27

**Authors:** Bo Qian, Chengqiang Wang, Zhen Zeng, Yuan Ren, Dayu Li, Jia-Le Song

**Affiliations:** ^1^Department of Nutrition and Food Hygiene, School of Public Health, Guilin Medical University, Guilin 541004, China; ^2^State Key Laboratory of Molecular Vaccinology and Molecular Diagnostics, School of Public Health, Xiamen University, Xiamen 361102, China; ^3^Department of Children and Maternal and Child Health, XianYa School of Public Health, Central South University, Changsha 410078, Hunan, China; ^4^Department of Parasitology, School of Basic Medicine, Guilin Medical University, Guilin 541004, China; ^5^Department of Surgery, School of Medicine, University of Maryland, Baltimore 21201, USA; ^6^Department of Nutrition and Gastrointestinal Surgery, The Second Affiliated Hospital of Guilin Medical University, Guilin 541004, China

## Abstract

Ulcerative colitis is a chronic gastrointestinal disease characterized by intestinal inflammation and serious mucosal damage. As a naturally hydroxycinnamic acid, sinapic acid (SA) has antioxidant, anticancer, and neuroprotective activities. We investigated the anticolitic effect and potential mechanisms of SA in DSS-induced colitis in Kunming (KM) mice. SA treatment significantly reduced body weight loss, colon shortening, and intestinal wall thickening in colitis mice. SA treatment also significantly reduced the histological infiltration of inflammatory cells and decreased myeloperoxidase (MPO) activity in the colons of colitis mice. The administration of SA attenuated oxidative damage by enhancing the activity of superoxide dismutase (SOD), glutathione peroxidase (GSH-Px), and catalase and reduced the serum and colonic mRNA levels of proinflammatory cytokines in colitis mice. We used qRT-PCR and Western blotting assays and demonstrated that SA reduced the activation of the NLRP3 inflammasome and attenuated intestinal permeability by enhancing the expression of ZO-1, occludin, and claudin-1 in colitis mice. Here, we conclude that SA exhibits great anticolitic activity against DSS-induced colitis by enhancing the activity of antioxidant enzymes, reducing intestinal inflammation, and maintaining the intestinal barrier. Finally, we suggest that SA may be a safe adjuvant for the prevention of clinical colitis.

## 1. Introduction

Both ulcerative colitis (UC) and Crohn's disease (CD) are serious gastrointestinal (GI) diseases, and they are classified as human inflammatory bowel diseases (IBDs). The occurrence of IBD has traditionally been the highest in North America and Western Europe, but it has also increased in Eastern Asia (including China and South Korea) [1, 2]. The etiology of IBD is still unclear, and it is well known that IBD is associated with various risk factors, such as environmental pollution, poor lifestyle, a Westernized diet, immunological disorders, and genetic susceptibility.

In general, IBD is a recursive and chronic GI disease that is characterized by intestinal inflammatory imbalance and serious mucosal tissue damage in the clinic. Elevated levels of proinflammatory cytokines (such as tumor necrosis factor-*α* (TNF-*α*), interferon-*γ* (IFN-*γ*), and interleukin- (IL-) 1*β*, IL-6, IL-8, and IL-17*α*) have been observed in human IBD patients [1, 3]. These overactivated proinflammatory cytokines induce the accumulation and infiltration of neutrophils and macrophages into colon tissue. Activated macrophages produce a potent mixture of broadly active inflammatory cytokines (TNF-*α*, IL-1*β*, and IL-6), generate reactive oxygen species (ROS), and activate the NLRP3 inflammasome to destroy the intestinal barrier and other colon tissue [4, 5]. Therefore, research on IBD therapy has focused on inhibiting the activation of inflammatory macrophages and reducing the proinflammatory cytokines that induce inflammatory cascades [6, 7].

Some chemical reagents, such as anti-inflammatory 5-aminosalicylic acid (5-ASA), corticosteroids, thiopurines, calcineurin inhibitors, a Janus kinase inhibitor (tofacitinib), and immunosuppressive agents (such as the anti-TNF-*α* monoclonal antibody infliximab and the anti-integrin *α*_4_*β*_7_ monoclonal antibody vedolizumab), have been widely used in clinical IBD therapy [8]. However, these reagents are also associated with serious complications and undesirable side effects, including systemic immunosuppression, kidney toxicity, diabetes, weight gain, and high blood pressure [8, 9].

On the other hand, complementary and alternative medicine (CAM) has become a hot research topic regarding therapy for IBD and other GI disorders [10]. Some natural herbal medicines in traditional Chinese medicine (TCM) and other dietary phytochemicals have been reported to have anti-inflammatory activity and attenuate IBD in animal studies and in human clinical trials [11]. Hence, the development of effective and safe therapeutic agents to treat IBD has become increasingly important. Regarding nutrition therapy, dietary interventions (such as supplementation with nutrients and phytochemicals and therapeutic anti-IBD diets) have helped to attenuate clinical symptoms and improve the quality of life of IBD patients [12].

Sinapic acid, also called 3,5-dimethoxy-4-hydroxycinnamic acid, is a naturally occurring hydroxycinnamic acid that is widely found in vegetables, fruits, cereals, and oilseed crops, as well as in wine and vinegar. Sinapic acid has been reported to have many biological activities, including antioxidant, anti-inflammatory, anticancer, antimutagenic, antiglycemic, neuroprotective, and antibacterial activities [13]. Recently, Lee reported that sinapic acid exhibits anti-inflammatory activity in 2,4,6-trinitrobenzenesulfonic acid- (TNBS-) induced colitis mice [14]. However, no study has investigated the anticolitic activity and potential mechanisms in dextran sulfate sodium- (DSS-) induced colitis in Kunming (KM) mice. Therefore, in this study, we investigated the anti-inflammatory effect of sinapic acid in a classic DSS-induced experimental mouse model.

## 2. Materials and Methods

### 2.1. Chemical Reagents

Sinapic acid (S106903, purity ≥ 98%) was purchased from Aladdin Chemicals Co., Ltd. (Shanghai, China). Dextran sulfate sodium (DSS, molecular weight: 36,00-50,000) was obtained from MP Biomedicals (Solon, OH, USA). TRIzol reagent, OligodT18 primer, murine Moloney leukemia virus (MMLV) reverse transcriptase, RNase, 2x SYBR Premix Ex Taq II, and 50x ROX reference dye inhibitor were purchased from Invitrogen Life Technologies (Carlsbad, CA, USA). All other reagents were of analytical grade and were purchased from Tokyo Chemical Industry (Shanghai) Chemicals Co., Ltd. (Shanghai, China).

### 2.2. Animal Grouping and Experimental Design

Specific pathogen-free- (SPF-) grade male Kunming (KM) mice (5-week-old, 13-15 g) were purchased from the Experimental Animal Center of Guilin Medical University (Guilin, Guangxi, China). KM mice first originated from Swiss mice are widely produced and are widely used in pharmacological studies in China [15]. All animals were housed in a standard environment (12 h light/dark cycle, 25°C ± 1°C) and had free access to a standard AIN-93G diet and fresh water. The induction of experimental colitis was performed as in our previous study [16, 17]. The animals were randomly divided into four groups of seven mice each: group A (normal control), in which the mice were treated with 0.9% normal saline; group B (control), in which the mice were administered DSS (2%, g/mL) for 7 days; and groups C and D, in which the mice received DSS (2%, g/mL) and were given low (10 mg/kg) or high (50 mg/kg) doses of sinapic acid (dissolved in 0.5% of carboxymethyl cellulose sodium solution) intragastrically (0.2 mL/mouse) for 7 days. At the end of the experiment, the mice were euthanized using CO_2_ and sacrificed. Colon length and colon weight were determined immediately. The animal protocol used in the present study was reviewed by the Institutional Animal Care and Use Committee of Guilin Medical University (approval number GLMC201703019).

### 2.3. Evaluation of the Disease Activity Index (DAI)

The DAI was used to evaluate the grade and extent of intestinal inflammation. Body weight, stool consistency, and the presence of blood in the stools were monitored daily to determine the DAI. Scores were determined as follows: body weight loss (0, none; 1, 1-5%; 2, 5-10%; 3, 10-20%; 4, >20%), diarrhea (0, normal stools; 2, loose stools; 4, watery diarrhea), and blood (0, normal; 2, slight bleeding; 4, gross bleeding) [17].

### 2.4. Evaluation of Colon Inflammation Scores

The inflammation scores of the colons were determined by two independent investigators blinded to treatment and according to the method described by Stillie and Stadnyk [18]. Scores were determined as follows: extent of oedema (1, present; 0, absent), crypt damage (0–5), infiltration of cells (0–5), and presence of ulcers (0–3). The scores for each parameter were added to obtain the total inflammation score, with a maximum value of 14.

### 2.5. Serum Preparation and Proinflammatory Cytokine Assays

For serum preparation, blood was first collected from the inferior vena cava from each animal using a vacuum blood collection tube (Hanbaihan Medical Devices Co. Ltd., Beijing, China). The collected blood samples were centrifuged using an Eppendorf 5424R high-speed centrifuge (3000 × g, 10 min) at 4°C to prepare serum samples. Serum levels of TNF-*α* (kt99985), IL-8 (kt21138), IL-4 (kt99969), and IL-10 (kt99847) were determined by commercial ELISA kits (MSK Biological Technology Co. Ltd., Wuhan, China), and the levels of IFN-*γ* (no. 430807), IL-1*β* (no. 437007), IL-6 (no. 431304), and IL-17*α* (no. 432507) were determined using commercial ELISA kits (BioLegend, Inc., San Diego, CA, USA) according to the manufacturer's instructions.

### 2.6. Evaluation of the Permeability of the Colonic Mucosa

To evaluate the permeability of the colonic mucosa, serum levels of LPS and fluorescein isothiocyanate-dextran 40 (FITC-D40, FD-40) were determined. The mice were not provided food or water for 4 h before sacrifice; FD-40 (60 mg/100 g) was administered once by oral gavage. Following this treatment, blood was collected as described in Section 2.5. The serum levels of FD-40 were determined using a FLUOstar OPTIMA fluorescence microplate reader (BMG Labtech GmbH, Ortenberg, Germany) with suitable assay conditions (excitation wavelength = 490 nm, emission wavelength = 520 nm). The serum levels of lipopolysaccharide (LPS) were measured using an ELISA kit (CSB-E13066m, CUSABIO Technology LLC, Wuhan, China) following the protocol.

### 2.7. Tissue Sample Collection and Histological Observations

Four tissue samples of the distal colon (approximately 5 cm) were collected from each animal and further subjected to histological observation. The colon samples were first washed with cold normal saline to remove blood and feces and then fixed with 10 mL of neutral buffered formalin solution (10%, *v*/*v*) for 24 h. The fixed tissue was dehydrated in ethanol, embedded in paraffin, and serially sectioned. Tissue sections (4 *μ*m thick) were then prepared and stained with hematoxylin and eosin (H&E). Histological images were acquired with a Leica DM4B microscope equipped with a Leica DFC550 CCD digital camera (Leica Microsystems Inc., Buffalo Grove, USA).

### 2.8. Measurement of Antioxidant-Related Biomarkers in Colon Tissue

The collected colon samples were washed with ice-cold saline. Then, the colon tissues (100 mg) were homogenized in phosphate-buffered saline (PBS) on ice. The colonic levels of myeloperoxidase (MPO, A044), glutathione (GSH, A006-1), superoxide dismutase (SOD, A001-3), glutathione peroxidase (GSH-Px, A005), catalase (CAT, A007-1-1), and malondialdehyde (MDA, A003-1) were all determined by commercial kits (Nanjing Jiancheng Biotechnology Co., Ltd., Nanjing, Jiangsu, China) according to the manufacturer's protocol.

### 2.9. Total RNA Extraction and Quantitative Real-Time Polymerase Chain Reaction (qRT-PCR)

Colon tissues were collected from all of the treated animals. After the tissues were washed with normal saline, total RNA was extracted using TRIzol reagent (Invitrogen, Carlsbad, CA, USA) according to the manufacturer's instructions. The extracted RNA (1 *μ*g) was mixed with a cocktail reagent containing OligodT18 (1 *μ*L), RNase (1 *μ*L), dNTP (1 *μ*L), MMLV enzymes (1 *μ*L), and 5x buffer (10 *μ*L) to synthesize cDNA (37°C for 120 min, 99°C for 4 min, and 4°C for 3 min). cDNA (2 *μ*L) was mixed with primer (10 *μ*mol/L, 2 *μ*L), 2x SYBR Premix Ex Taq II (10 *μ*L), 50x ROX reference dye (0.4 *μ*L), and ddH_2_O (5.6 *μ*L), and PCR was performed in an automatic thermocycler (QuantStudio™ 6 Flex PCR, Life Technologies, Gaithersburg, MD, USA) for 40 cycles at 94°C for 15 sec, 60°C for 60 sec, and 72°C for 50 sec, followed by 10 min at 75°C. The relative transcription levels of the mRNAs were calculated according to the 2*ΔΔ*Ct formula (ΔΔCt = average of ΔCt control − ΔCt treated). The sequences of the primers used for qRT-PCR are provided in Table 1.

### 2.10. Protein Extraction and Western Blot Analysis

Colon tissues were collected from all of the animals and washed with ice-cold normal saline. Total protein samples were extracted using radioimmunoprecipitation assay (RIPA) buffer (Beyotime Biotechnology Co., Shanghai, China) on ice, centrifuged at 13,000 × g for 30 min at 4°C, and denatured by boiling (98°C). The protein concentrations of the extracted samples were determined using a Quartzy Quick Start Bradford Protein Assay Kit (Bio-Rad Laboratories, Hercules, Calif., USA). Total protein (20~50 *μ*g) was separated on Mini-PROTEAN precast gels (4-20% SDS-PAGE, Bio-Rad) for 1.5 h and transferred to nitrocellulose membranes. The membranes were sealed for 2 h in sealing fluid, washed 3 times with PBS containing 0.1% Tween 20 (PBS-T), and incubated with primary antibodies against occludin (sc-133256, Santa Cruz Biotechnology, Inc., Dallas, TX, USA), claudin-1 (sc-293233; Santa Cruz), zonula occludens- (ZO-) 1 (AF0231, Beyotime), NLRP3 (AF2155; Beyotime), IL-1*β* (sc-32294; Santa Cruz), caspase-1 (AF1681, Beyotime), ASC (sc-514559; Santa Cruz), and GAPDH (sc-47724; Santa Cruz) for 60 min at 37°C. After washing with PBS-T, the blots were incubated with horseradish peroxidase-conjugated mouse anti-rabbit IgG secondary antibody (no. 45262, Cell Signaling Technology Inc., USA) for 40 min at 37°C. Following washing three times with PBS-T, the blots were incubated with enhanced chemiluminescence (ECL) HRP substrates (Millipore, Billerica, MA, USA) and visualized with an iBright FL1000 system (Invitrogen). The densitometry analysis of the bands was performed using ImageJ (NIH Image, Bethesda, MD, USA).

### 2.11. Statistical Analysis

The experimental data are presented as the mean ± standard deviation (SD) of three replicates. The differences between the mean values of each group were assessed by one-way ANOVA with Dunnett's test. *p* < 0.05 was considered an indication of a statistically significant difference. The SPSS 21.0 statistical software package (SPSS Inc., Chicago, IL, USA) was used for the analysis.

## 3. Results

### 3.1. Effects of Sinapic Acid on the Clinical Observations of DSS-Induced Colitis Mice

**Figure 1:**
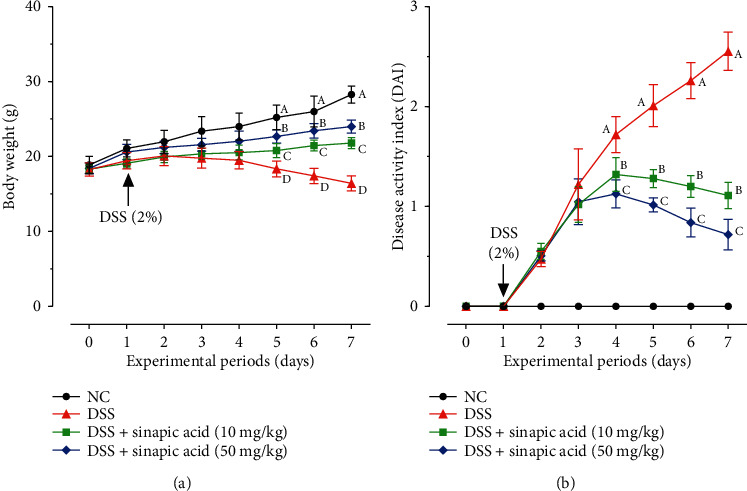
Effect of sinapic acid on (a) body weight changes and (b) disease activity index (DAI) in dextran sulfate sodium- (DSS-) induced colitis mice. The data are presented as the mean ± SD (*n* = 7 mice/group). NC indicates normal control. ^A–D^Bars with different superscript letters are significantly different (*p* < 0.05) according to Duncan's multiple range test.

The effect of sinapic acid on the clinical observations of DSS-induced colitis in KM mice is presented in Figure 1(a). The body weight gain of the mice in the DSS group was significantly lower than that of the normal mice (*p* < 0.05). However, sinapic acid treatment exhibited preventive activity and reduced the DSS-induced body weight loss in colitis mice. In addition, serious symptoms, such as diarrhea, gross bleeding, and body weight loss (indicated by increased DAI), were observed in DSS-treated colitis mice. As shown in Figure 1(b), the administration of 10 mg/kg and 50 mg/kg of sinapic acid reduced the increased DAI scores in colitis mice.

### 3.2. Effects of Sinapic Acid on the Colon Length and the Colon Weight to Length Ratio of DSS-Induced Colitis Mice

**Figure 2:**
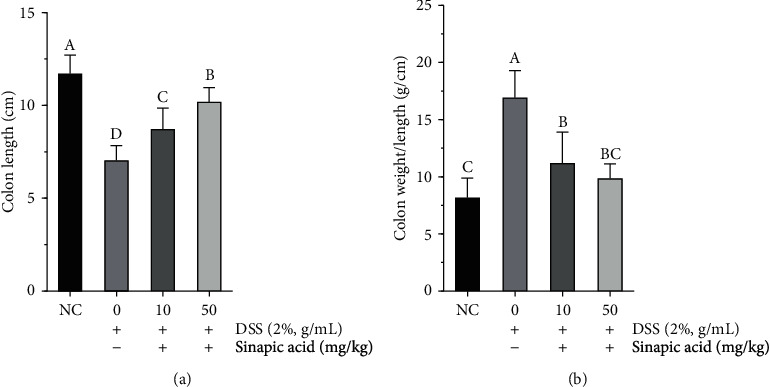
Effect of sinapic acid on (a) the colon length and (b) the colon weight to length ratio in dextran sulfate sodium- (DSS-) induced colitis mice. The data are presented as the mean ± SD (*n* = 7 mice/group). NC indicates normal control. ^A–D^Bars with different superscript letters are significantly different (*p* < 0.05) according to Duncan's multiple range test.

Both colon length shortening and colon weight increases were typical symptoms in DSS-induced colitis mice [19]. As presented in Figure 2(a), the administration of DSS significantly decreased colon length in colitis mice (to 7.04 ± 0.74 cm), and the colons were shorter than those of normal mice (11.71 ± 0.92 cm). Sinapic acid treatment (10 mg/kg and 50 mg/kg) increased the colon length to 8.73 ± 1.04 cm and 10.20 ± 0.70 cm, respectively. In addition, sinapic acid also significantly reduced the colon weight to length ratio in colitis mice (Figure 2(b)). In addition, both low dose and high dose of sinapic acid were able to reduce the colon weight to length ratio. However, there is no significant difference between the low dose and high dose of sinapic acid-treated colitis mice (*p* > 0.05).

### 3.3. Effect of Sinapic Acid on Histology in DSS-Induced Colitis Mice

**Figure 3:**
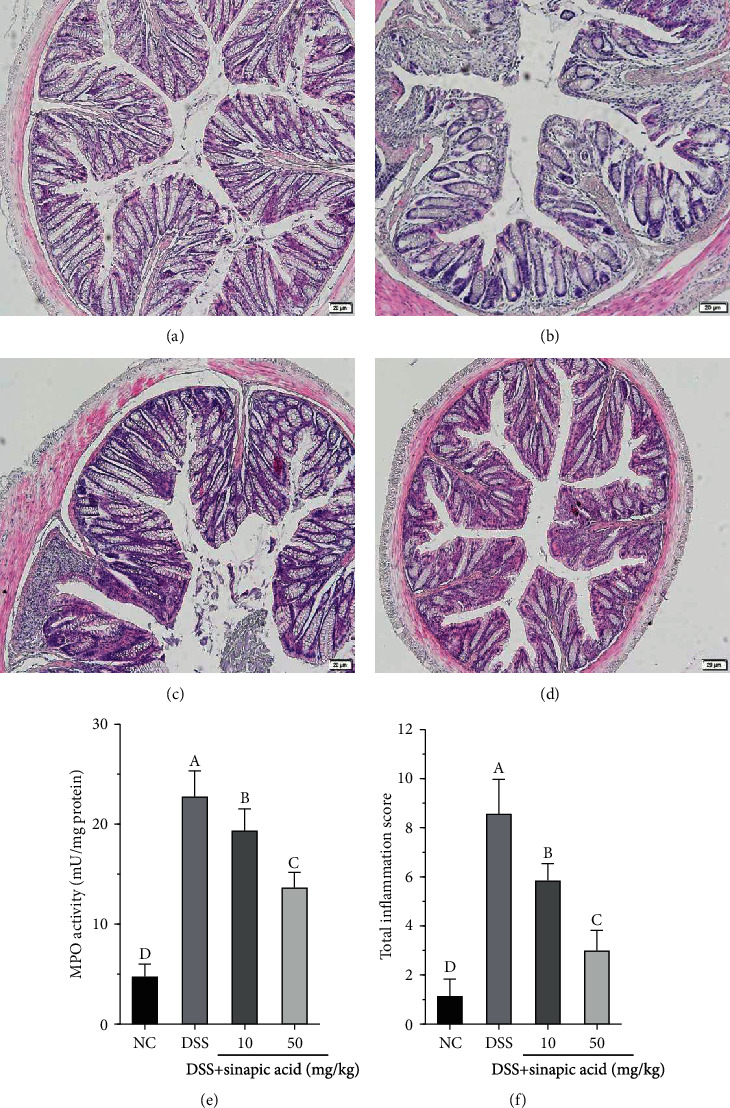
Effect of sinapic acid on histology observations (10x magnification) in dextran sulfate sodium- (DSS-) induced colitis mice: (a) normal control (NC); (b) DSS-treated colitis mice; (c, d) colitis mice treated with a low dose of sinapic acid (10 mg/kg) and a high dose of sinapic acid (50 mg/kg); (e) MPO activity; (f) total inflammation score. The data are presented as the mean ± SD (*n* = 7 mice/group). ^A–D^Bars with different superscript letters are significantly different (*p* < 0.05) according to Duncan's multiple range test.

To evaluate the anticolitic effect of sinapic acid in DSS-induced colitis mice, histological observations were performed by staining for H&E. As shown in Figure 3(a), tissue sections from normal mice showed intact surface epithelium, cryptal glands, stroma, and submucosa. However, DSS treatment caused significant disruption of the crypt epithelium and extensive mucosal damage with a large number of inflammatory cells (Figure 3(b)) and increased MPO activity and inflammation scores (Figures 3(e) and 3(f)). As shown in Figures 3(c) and 3(d), tissue sections from sinapic acid-treated colitis mice had more intact surface epithelium and crypt glands and lower levels of inflammation than colitis mice. Sinapic acid treatment also significantly decreased MPO accumulation and reduced inflammation scoring in colitis mice (Figures 3(c) and 3(d)).

### 3.4. Effect of Sinapic Acid on Colonic Levels of Superoxide Dismutase (SOD), Glutathione Peroxidase (GSH-Px), Catalase, Glutathione (GSH), and Malondialdehyde (MDA) in DSS-Induced Colitis Mice

Many studies have observed serious oxidative stress-induced tissue damage in colitis and colitis associated with colon cancer in human and rodent studies [20]. In this study, we found that DSS treatment significantly induced oxidative stress in the colons of colitis mice. As shown in Table 2, DSS significantly decreased the activities of endogenous antioxidant enzymes, including SOD, GSH-Px, catalase, and GSH, in the colon tissue of colitis mice. Following treatment with sinapic acid for 7 days, the levels of colonic antioxidant enzymes (SOD, GHS-Px, and catalase) and the nonenzymatic antioxidant GSH were significantly increased compared with those in DSS-treated colitis mice. In addition, we also found that, compared with 10 mg/kg sinapic acid, 50 mg/kg sinapic acid increased the levels of these antioxidants.

Furthermore, the administration of DSS significantly induced the generation of MDA in the colons of colitis mice (to 4.59 ± 0.72 nmol/mg protein) compared with normal mice (0.34 ± 0.11 nmol/mg protein). However, following with the administration of 10 mg/kg and 50 mg/kg sinapic acid, the MDA levels were reduced to 3.26 ± 0.82 nmol/mg protein and 2.14 ± 0.76 nmol/mg protein, respectively. In addition, sinapic acid also attenuated the DSS-induced reduction in colonic GSH levels in the colitis mice. The colonic GSH levels in the colitis mice significantly increased following treatment with sinapic acid, with the increased levels ranging from 1.45 ± 0.57 to 1.83 ± 0.30 *μ*mol/mg protein after treatment with 10 and 50 mg/kg sinapic acid, respectively.

### 3.5. Effect of Sinapic Acid on Serum Levels of Proinflammatory Cytokines in DSS-Induced Colitis Mice

**Figure 4:**
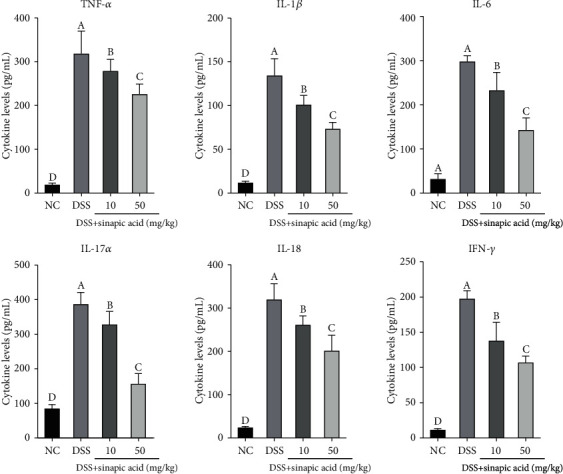
Effect of sinapic acid on the serum levels of proinflammatory cytokines (TNF-*α*, IL-1*β*, IL-6, IL-17*α*, IL-18, and IFN-*γ*) in dextran sulfate sodium- (DSS-) induced colitis mice. NC indicates normal control. The data are presented as the mean ± SD (*n* = 7 mice/group). ^a–d^Bars with different superscript letters are significantly different (*p* < 0.05) according to Duncan's multiple range test.

Many studies have suggested that increased proinflammatory cytokines are associated with the pathological process of IBD [7, 21]. As shown in Figure 4, mice treated with DSS showed high levels of proinflammatory cytokines (TNF-*α*, IL-1*β*, IL-6, IL-8, IL-17*α*, and IFN-*γ*) in the serum. However, the administration of low and high doses of sinapic acid reduced the generation of these inflammatory cytokines in colitis mice.

### 3.6. Effect of Sinapic Acid on the Serum Levels of Anti-Inflammatory Cytokines in DSS-Induced Colitis Mice

**Figure 5:**
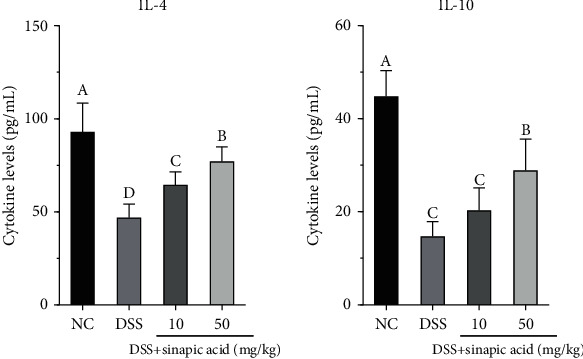
Effect of sinapic acid on the serum levels of anti-inflammatory cytokines (IL-4 and IL-6) in dextran sulfate sodium- (DSS-) induced colitis mice. NC indicates normal control. The data are presented as the mean ± SD (*n* = 7 mice/group). ^a–d^Bars with different superscript letters are significantly different (*p* < 0.05) according to Duncan's multiple range test.

Promoting the generation of some anti-inflammatory cytokines (such as IL-4 and IL-10) has been shown to attenuate colitis *in vivo* [22]. In this study, the serum levels of anti-inflammatory IL-4 and IL-10 were determined by an ELISA. As shown in Figure 5, DSS treatment significantly decreased the levels of IL-4 and IL-10 in the serum of the colitis mice. The administration of sinapic acid increased the serum levels of IL-4 and IL-10 in colitis mice. Compared with 10 mg/kg sinapic acid, a dose of 50 mg/kg sinapic acid significantly increased the serum levels of IL-4 and IL-10 (*p* < 0.05).

### 3.7. Effect of Sinapic Acid on Colonic mRNA Levels of Inflammation-Related Cytokines in DSS-Induced Colitis Mice

**Figure 6:**
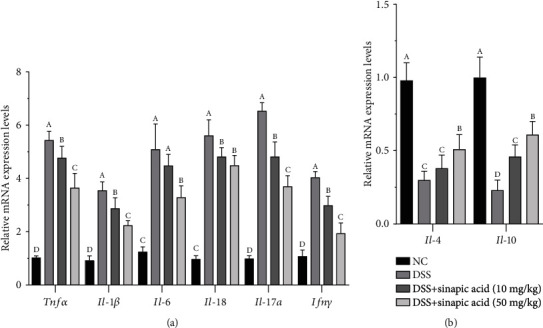
Effect of sinapic acid on the serum levels of anti-inflammatory cytokines (IL-4 and IL-6) in dextran sulfate sodium- (DSS-) induced colitis mice. NC indicates normal control. The data are presented as the mean ± SD (*n* = 7 mice/group). ^A–D^Bars with different superscript letters are significantly different (*p* < 0.05) according to Duncan's multiple range test.

We used qRT-PCR assay to further understand the effect of sinapic acid on the regulation of proinflammatory and anti-inflammatory cytokine generation in the colons of colitis mice. As shown in Figure 6(a), DSS significantly increased the mRNA expression of proinflammatory cytokines (including TNF-*α*, IL-1*β*, IL-6, IL-18, IL-17*α*, and IFN-*γ*) in the colons of colitis mice. Compared with 10 mg/kg sinapic acid, the administration of 50 mg/kg sinapic acid significantly reduced the mRNA levels of some cytokines (TNF-*α*, IL-1*β*, IL-6, and IL-17*α*). However, there was no significant difference in the mRNA levels of IL-18 in 10 mg/kg and 50 mg/kg sinapic acid-treated colitis mice (*p* > 0.05). In addition, both 10 mg/kg and 50 mg/kg sinapic acid significantly increased the mRNA levels of IL-4 and IL-10 in the colons of colitis mice (Figure 6(b)). However, there was no significant difference in IL-4 mRNA expression in sinapic acid (10 mg/kg) and DSS-treated colitis mice (*p* > 0.05).

### 3.8. Effect of Sinapic Acid on Serum Levels of LPS and FD-40 in DSS-Induced Colitis Mice

**Figure 7:**
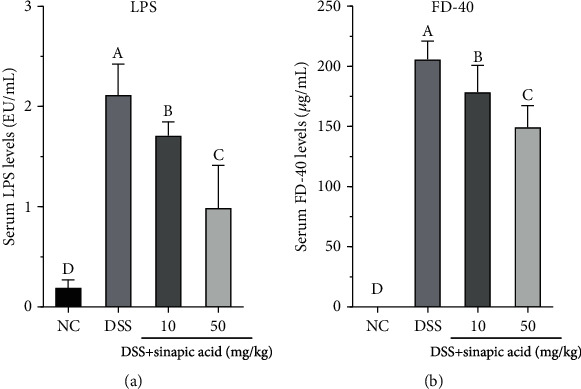
Effect of sinapic acid on serum levels of LPS and FD-40 in dextran sulfate sodium- (DSS-) induced colitis mice: (a) serum levels of LPS; (b) serum levels of FD-40. NC indicates normal control. The data are presented as the mean ± SD (*n* = 7 mice/group). ^A–D^Bars with different superscript letters are significantly different (*p* < 0.05) according to Duncan's multiple range test.

As shown in Figure 7, DSS treatment significantly induced the serum levels of LPS and FD-40 in the colitis mice. The administration of sinapic acid (both 10 mg/kg and 50 mg/kg) reduced the serum levels of LPS in colitis mice. The administration of 50 mg/kg of sinapic acid more strongly reduced serum LPS levels than 10 mg/kg sinapic acid. We also observed reduced serum levels of FD-40 in sinapic acid-treated colitis mice (Figure 7(b)).

### 3.9. Effect of Sinapic Acid on Colonic mRNA Levels of Claudin-1, Occludin, and ZO-1 in DSS-Induced Colitis Mice

**Figure 8:**
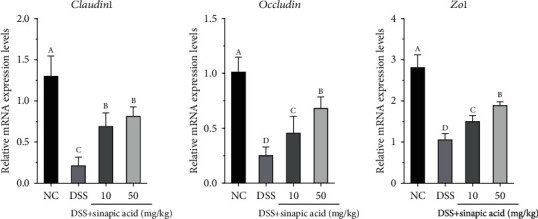
Effect of sinapic acid on colonic mRNA levels of claudin-1, occludin, and ZO-1 in dextran sulfate sodium- (DSS-) induced colitis mice. NC indicates normal control. The data are presented as the mean ± SD (*n* = 7 mice/group). ^a–d^Bars with different superscript letters are significantly different (*p* < 0.05) according to Duncan's multiple range test.

In addition, the mRNA levels of some tight junction- (TJ-) related factors, such as claudin-1, occludin, and ZO-1, were determined using the qRT-PCR assay. As shown in Figure 8, DSS treatment significantly decreased the colon mRNA levels of claudin-1, occludin, and ZO-1 compared with those of normal mice. However, sinapic acid treatment was able to increase the mRNA expression of claudin-1, occludin, and ZO-1 in the colons of the colitis mice. Compared with 10 mg/kg sinapic acid, 50 mg/kg sinapic acid significantly increased the mRNA expressions of occludin and ZO-1 (*p* < 0.05).

### 3.10. Effect of Sinapic Acid on Colonic Protein Levels of Claudin-1, Occludin, and ZO-1 in DSS-Induced Colitis Mice

**Figure 9:**
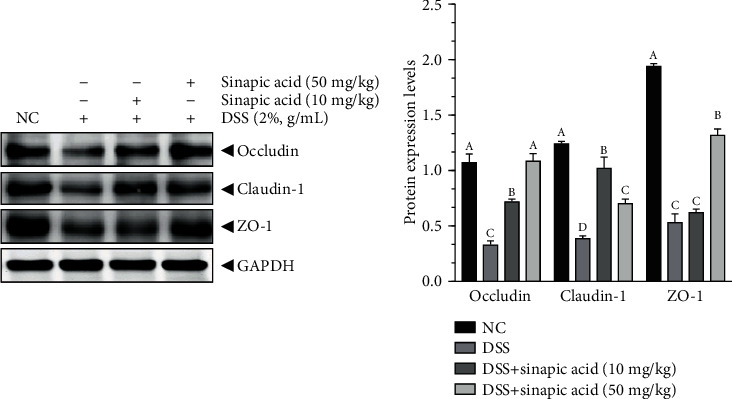
Effect of sinapic acid on the colonic protein levels of claudin-1, occludin, and ZO-1 in dextran sulfate sodium- (DSS-) induced colitis mice. The data are presented as the mean ± SD. NC indicates normal control. ^a–d^Bars with different superscript letters are significantly different (*p* < 0.05) according to Duncan's multiple range test.

We also investigated the effect of sinapic acid on the protein levels of claudin-1, occludin, and ZO-1 in the colon of the DSS-induced colitis mice. As shown in Figure 9, sinapic acid treatment also increased the protein levels of claudin-1, occludin, and ZO-1 in the colons of the colitis mice (*p* < 0.05). However, the low dose of sinapic acid (10 mg/kg) more strongly increased the protein levels of claudin-1 than 50 mg/kg sinapic acid (*p* < 0.05).

### 3.11. Effect of Sinapic Acid on the Protein Levels of NLRP3 Inflammasome in DSS-Induced Colitis Mice

**Figure 10:**
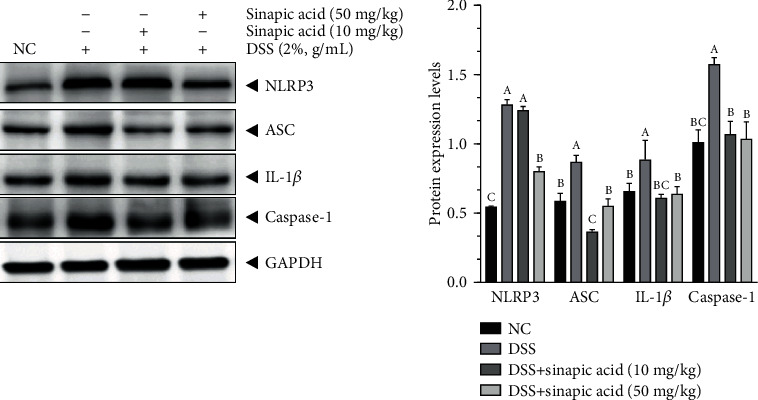
Effect of sinapic acid on the NLRP3 inflammasome in dextran sulfate sodium- (DSS-) induced colitis mice. The data are presented as the mean ± SD. NC indicates normal control. ^a–d^Bars with different superscript letters are significantly different (*p* < 0.05) according to Duncan's multiple range test.

As shown in Figure 10, DSS treatment significantly induced increases in the protein levels of NLRP3, ASC, IL-1*β*, and caspase-1 in the colons of the colitis mice. Following treatment with 10 mg/kg and 50 mg/kg sinapic acid, the protein levels of NLRP3, ASC, IL-1*β*, and caspase-1 were reduced in colitis mice. The administration of 50 mg/kg sinapic acid more strongly reduced the protein levels of NLRP3 in the colon than 10 mg/kg sinapic acid (*p* < 0.05). In addition, 10 mg/kg sinapic acid more strongly reduced the protein levels of ASC and IL-1*β* in the colon than 50 mg/kg of sinapic acid. However, there were no significant differences in the colonic protein levels of caspase-1 in 10 and 50 mg/kg sinapic acid-treated colitis mice.

## 4. Discussion

Inflammatory bowel disease (IBD) is a serious gastrointestinal disease that is common in North America, Europe, and some Asian countries [2]. In recent years, the morbidity of IBD in China has also increased. Traditional anti-inflammatory, immunosuppressant, and glucocorticoid treatments have been used as basic therapies for clinical IBD. However, these traditional treatments are associated with undesirable side effects and high treatment costs. Recently, traditional herbal medicines have become alternative therapies to conventional therapies for the treatment of IBD. Many studies and review articles have indicated that herbal medicines exhibit robust activity against IBD [10, 11, 23–28]. Moreover, the popularity of traditional Chinese medicine among patients with IBD has rapidly increased not only in Asia but also in the Western Hemisphere [10].

In this study, we investigated the anticolitic effect of sinapic acid using DSS-induced mouse colitis. Sinapic acid (10 and 50 mg/kg) and DSS (2%) were orally administered to mice, and then, clinical colitis was assessed by examining body weight loss, shortening of the colon length, the colon weight to length ratio, and the DAI. Treatment with 2% DSS induced a significant decrease in body weight, shortening of the colon, and serious DAI in colitis mice. However, sinapic acid administration attenuated body weight loss, colon shortening, and intestinal wall thickening and decreased the DAI in DSS-induced colitis mice. Upon histological observation, it was found that DSS treatment significantly increased inflammatory cell infiltration into the colon tissues, directly induced mucosal erosion and damage, and caused the distortion and loss of crypts. In addition, DSS treatment also caused increased inflammatory scores. However, sinapic acid treatment was able to reduce the number of inflammatory cells (such as neutrophils and macrophages) that infiltrated into the colon tissue and attenuated the inflammatory reaction-induced tissue damage and crypt loss. These results were similar to those in Lee's report [14].

MPO is a classic biomarker used to evaluate the levels of inflammatory reactions in the colon. MPO, which is abundantly expressed in neutrophils and to a lesser extent in monocytes and certain types of macrophages, belongs to the heme peroxidase-cyclooxygenase superfamily. It is a specific marker that may be used to determine neutrophil influx into the colon tissue. In this study, we found that DSS significantly increased the colonic levels of MPO in colitis mice. This result suggests that the overaccumulation of inflammatory cells results in infiltration into the colon tissue. However, the administration of sinapic acid significantly reduced the generation of MPO in the colons of colitis mice. The decrease in MPO activity may be explained by a reduction in neutrophil accumulation in inflamed tissues. Sinapic acid has been reported to significantly reduce colonic MDA generation in TNBS-induced colitis mice [14]. The results suggest that sinapic acid markedly reduces leukocyte (neutrophil and macrophage) infiltration and decreases the colonic MPO levels to ameliorate inflammatory conditions in the colon tissues of DSS-induced colitis mice. IBD is a chronic inflammatory disease that is associated with the overaccumulation of inflammatory cells in the colon tissue. The infiltration of inflammatory cells is an important source of reactive oxygen species (ROS) and reactive nitrogen species (RNS), which act as cytotoxic agents by cross-linking proteins, lipids, and nucleic acids, thus causing cell damage [29]. In general, the overproduction of ROS and RNS strongly disrupts the oxidant/antioxidant balance, as shown by increased lipid peroxidation and a reduction in colonic GSH content. In both humans and animals, elevated levels of ROS-induced oxidative stress play an important role in the process of IBD-associated intestinal inflammation and mucosal damage [30]. ROS-induced oxidative stress also induces lipid peroxidation and DNA and tissue damage, destroys the intestinal mucosal barrier, and enhances intestinal permeability [31]. Under normal physiological conditions, the endogenous antioxidant system has key activity against free radicals and ROS-induced oxidative damage [32]. Enzymatic SOD, GSH-Px, catalase, and nonenzymatic GSH are able to scavenge free radicals and ROS and prevent oxidative stress-induced lipid peroxidation and cell damage. In this study, we found that, compared with no treatment, sinapic acid treatment significantly increased the levels of SOD, GSH-Px, catalase, and GHS in colitis mice. Both MDA and 4-hydroxynonenal (4-HNE) are endogenous lipid peroxidation products and are typical biomarkers of ROS-induced oxidative stress. DSS-induced elevations in the generation of MDA in the colons of colitis mice were observed in this study. Following administration, sinapic acid was able to reduce the colonic generation of MDA in colitis mice, and this result agreed with that of a study by Lee [14]. This evidence suggests that sinapic acid, which has antioxidant activity against oxidative stress, induces colon tissue damage in colitis mice.

It is well accepted that the overproduction of proinflammatory cytokines (such as TNF-*α*, IL-1*β*, IL-6, IL-17*α*, and IFN-*γ*) causes serious tissue damage, enhances inflammatory reactions in human IBD, and is observed in chemically induced experimental rodent colitis models (including DSS- and TNBS-induced colitis) [3, 6, 7, 19]. Therefore, blocking and reducing proinflammatory cytokines are the main target of treatment for IBD clinical therapy. Recently, immunological therapy targeting anti-TNF-*α* activity was successful in attenuating clinical symptoms and promoting mucosal healing in IBD patients [33]. In addition, other inflammatory cytokines, such as IL-6 and IL-17*α*, also show strong activity in promoting the progression of IBD. Clinical studies have reported elevated serum levels of IL-1*β*, IL-6, and IL-17*α* in human IBD patients [21, 34, 35]. Elevated IL-1*β* may induce intestinal permeability, activate inflammatory cells, and enhance the accumulation of IL-17*α* in Th17 cells [36]. Blocking the activation of IL-1*β* attenuates the symptoms of DSS-induced colitis and reduces IL-6 gene expression [37].

In this study, we found that the serum levels of TNF-*α*, IL-1*β*, IL-6, IL-8, IL-17*α*, and IFN-*γ* in colitis mice were significantly decreased by treatment with sinapic acid. We also observed that sinapic acid treatment significantly reduced the mRNA expression levels of TNF-*α*, IL-1*β*, IL-6, IL-8, IL-17*α*, and IFN-*γ* in the colons of colitis mice. IL-4 and IL-10 are immunoregulatory cytokines with anti-inflammatory activity, and they exhibit the ability to attenuate clinical symptoms in IBD patients [22]. Sinapic acid treatment increased the serum levels of IL-4 and IL-10 in colitis mice and promoted the mRNA expression of these anti-inflammatory cytokines in the colons of colitis mice. The administration of IL-4 was able to prevent chemical-induced colitis in mice [38]. These results indicate that sinapic acid exhibits anti-inflammatory effects on DSS-induced colitis by modulating the balance of proinflammatory cytokines/anti-inflammatory cytokines.

Recently, it was shown that the NLRP3 inflammasome, which is a complex that contains NLRP3, ASC, and caspase-1, plays an important role in colitis and colitis-associated colon cancer (CAC) [5] and is also known as a potential target for the prevention of colitis and CAC [4]. In general, activated NLRP3 recruits the adaptor protein ASC and then promotes the transformation of procaspase-1 to caspase-1, which has strong biological activity to enhance the secretion of the proinflammatory cytokines IL-1*β* and IL-18 [5]. Here, we found that DSS significantly increased the serum and colonic levels of IL-1*β* and IL-18 in colitis mice. However, sinapic acid treatment also decreased the serum and colonic levels of IL-1*β* and IL-18, which were associated with decreased NLRP3, ASC, and caspase-1 protein levels in the colons of colitis mice. Reducing the activation of the NLRP3 inflammasome was able to inhibit inflammatory reactions and attenuated inflammatory injury in the colons of rodents with chemically induced experimental colitis [39]. In addition, Han et al. also reported the sinapic acid treatment showed an activity to inhibit the activation of NLRP3 inflammasome (by reducing the protein levels of NLRP3, ASC, caspase-1, and IL-1*β*) in rats with diabetic atherosclerosis [40].

It is well known that the intestinal epithelial barrier, which functions in nutrient absorption, plays an important role against the induction of damage by harmful exogenous substances (such as microorganisms, toxins, and antigens) during the maintenance of the normal function of the intestinal system under normal physiological conditions. To evaluate intestinal permeability, we determined the serum levels of LPS and FD-40 in sinapic acid-treated colitis mice. The administration of sinapic acid reduced the serum levels of LPS and FD-40 in colitis mice. The decreased levels of LPS and FD-40 indicated that the high intestinal permeability induced by DSS was reduced by sinapic acid treatment in colitis mice. TJ proteins, such as occludin, ZO-1, and claudins, are the main components of the intestinal barrier and are essential for cell-cell adhesion [41]. The normal levels of these TJ proteins play an important role in maintaining and regulating the function of the intestinal epithelial barrier. ZO-1 can connect to the cytoskeleton and is also connected to the intracellular loops of claudins and occludin [41]. The cytoskeleton-TJ structure is able to prevent pathogenic bacterial adherence or invasion into cells and reduce intestinal permeability during the progression of colitis [42]. In DSS-induced colitis mice, both the mRNA and protein levels of occludin, ZO-1, and claudin-1 were found to be decreased in the colon tissue [43]. However, our results showed that the mRNA and protein levels of occludin, ZO-1, and claudin-1 were significantly increased by sinapic acid treatment, which suggests that sinapic acid may maintain intestinal barrier function and reduce intestinal permeability through the regulation of TJ expression.

## 5. Conclusions

In conclusion, the present study demonstrated the potential anticolitic effects of sinapic acid in DSS-induced colitis KM mice. These results suggest that sinapic acid administration is able to reduce DSS-induced body weight loss, colonic shortening, and colitis-associated intestinal wall thickening. Sinapic acid treatment also reduced the infiltration and accumulation of inflammatory cells and reduced MPO activity in the colon tissue. In addition, sinapic acid also exhibited strong activity against increases in oxidative stress-related MDA generation and enhanced the activities of endogenous antioxidant enzymes and GSH in the colon tissue of colitis mice. In contrast, sinapic acid was shown to reduce the generation and mRNA expression levels of some inflammatory cytokines (TNF-*α*, IL-1*β*, IL-6, IL-8, IL-17*α*, and IFN-*γ*) and reduce the activation of the NLRP3 inflammasome in the colons of colitis mice. It was also observed that sinapic acid also enhanced the protein levels of occludin, ZO-1, and claudin-1 to maintain the normal function of the intestinal barrier in DSS-induced colitis in mice. Taken together, the results of this study clearly suggest that the potential mechanism of sinapic acid involves suppressing the production of inflammatory cytokines, improving antioxidant enzymes, reducing the activation of NLRP3, and maintaining the normal function of the intestinal barrier and that sinapic acid may be considered an important anticolitic agent against colon inflammatory disease.

## Figures and Tables

**Table 1:** The primer sequences used in this study for qRT-PCR.

Primer name	Forward (5′→3′)	Reverse (3′→5′)
Mouse *Tnfα*	ATGAGCACAGAAAGCATGA	AGTAGACAGAAGAGCGTGGT
Mouse *Il1β*	AATGAAAGACGGCACACCCA	TGCTTGTGAGGTGCTGATGT
Mouse *Il6*	CCTCTGGTCTTCTGGAGTACC	ACTCCTTCTGTGACTCCAGC
Mouse *Il4*	GGTCTCAACCCCCAGCTAGT	CCGATGATCTCTCTCAAGTGAT
Mouse *Il18*	AAACCACCGGAAGGAACCAT	CTTCACACAGAGCTGCAGAAA
Mouse *Il10*	GCTCTTACTGACTGGCATGAG	CGCAGCTC TAGGAGCATGTG
Mouse *Il17a*	TCTCCACCGCAATGAAGACC	CACACCCACCAGCATCTTCT
Mouse *Ifnγ*	TTCTTCAGCAACAGCAAGGC	TCAGCAGCGACTCCTTTTCC
Mouse *Claudin1*	ACGGCTCCGTTTTCTAGATGCC	CGTTTGGCTGCTGCTCTTGC
Mouse *Zo1*	CCATCTTTGGACCGATTGCTG	TAATGCCCGAGCTCCGATG
Mouse *Occludin*	ACGGTCCTCCTGGCTCAGTT	GATAAGCGAACCTTGGCGGC
Mouse *Gapdh*	CGGAGTCAACGGATTTGGTC	AGCCTTCTCCATGGTCGTGA

**Table 2:** Effect of sinapic acid on the colonic levels of superoxide dismutase (SOD), glutathione peroxidase (GSH-Px), catalase, glutathione (GSH), and malondialdehyde (MDA) in DSS-induced colitis mice.

Groups	SOD (U/mg protein)	GSH-Px (U/mg protein)	Catalase (U/mg protein)	GSH (*μ*mol/mg protein)	MDA (nmol/mg protein)
NC	11.89 ± 1.66^a^	6.44 ± 1.13^a^	1.88 ± 0.56^a^	3.76 ± 0.72^a^	0.34 ± 0.11^d^
DSS	2.55 ± 0.88^d^	2.05 ± 0.49^c^	0.69 ± 0.33^b^	0.87 ± 0.39^c^	4.59 ± 0.72^a^
DSS+SA (10 mg/kg)	5.79 ± 1.29^c^	4.43 ± 1.50^b^	0.81 ± 0.34^b^	1.45 ± 0.57^bc^	3.26 ± 0.82^b^
DSS+SA (50 mg/kg)	9.66 ± 1.44^b^	6.05 ± 1.33^a^	1.15 ± 0.43^b^	1.83 ± 0.30^b^	2.14 ± 0.76^c^

The data are presented as the mean ± SD(*n* = 7 mice/group). ^a–d^Values with different superscript letters are significantly different (*p* < 0.05) according to Duncan's multiple range test.

## Data Availability

All generated and analyzed data used to support the findings of this study are included within the article.
